# Modular characterization of SARS-CoV-2 nucleocapsid protein domain functions in nucleocapsid-like assembly

**DOI:** 10.1186/s43556-023-00129-z

**Published:** 2023-05-22

**Authors:** Yan Wang, Xiaobin Ling, Chong Zhang, Jian Zou, Bingnan Luo, Yongbo Luo, Xinyu Jia, Guowen Jia, Minghua Zhang, Junchao Hu, Ting Liu, Yuanfeiyi Wang, Kefeng Lu, Dan Li, Jinbiao Ma, Cong Liu, Zhaoming Su

**Affiliations:** 1 The State Key Laboratory of Biotherapy, Frontiers Medical Center of Tianfu Jincheng Laboratory, National Clinical Research Center for Geriatrics and Department of Geriatrics, West China Hospital, Sichuan University, Chengdu, 610044 Sichuan China; 2 State Key Laboratory of Genetic Engineering, Collaborative Innovation Center of Genetics and Development, Department of Biochemistry and Biophysics, School of Life Sciences, Fudan University, Shanghai, 200438 China; 3 College of Polymer Science and Engineering, Sichuan University, Chengdu, 610065 Sichuan China; 4 Bio-X Institutes, Key Laboratory for the Genetics of Developmental and Neuropsychiatric Disorders, Ministry of Education, Shanghai Jiao Tong University, Shanghai, 200030 China; 5 Interdisciplinary Research Center On Biology and Chemistry, Shanghai Institute of Organic Chemistry, Chinese Academy of Sciences, Shanghai, 201210 China; 6 State Key Laboratory of Bio-Organic and Natural Products Chemistry, Shanghai Institute of Organic Chemistry, Chinese Academy of Sciences, Shanghai, 200032 China

**Keywords:** Nucleocapsid protein, Filamentous assembly, Liquid–liquid phase separation

## Abstract

SARS-CoV-2 and its variants, with the Omicron subvariant XBB currently prevailing the global infections, continue to pose threats on public health worldwide. This non-segmented positive-stranded RNA virus encodes the multi-functional nucleocapsid protein (N) that plays key roles in viral infection, replication, genome packaging and budding. N protein consists of two structural domains, NTD and CTD, and three intrinsically disordered regions (IDRs) including the N_IDR_, the serine/arginine rich motif (SR_IDR_), and the C_IDR_. Previous studies revealed functions of N protein in RNA binding, oligomerization, and liquid–liquid phase separation (LLPS), however, characterizations of individual domains and their dissected contributions to N protein functions remain incomplete. In particular, little is known about N protein assembly that may play essential roles in viral replication and genome packing. Here, we present a modular approach to dissect functional roles of individual domains in SARS-CoV-2 N protein that reveals inhibitory or augmented modulations of protein assembly and LLPS in the presence of viral RNAs. Intriguingly, full-length N protein (N_FL_) assembles into ring-like architecture whereas the truncated SR_IDR_-CTD-C_IDR_ (N_182-419_) promotes filamentous assembly. Moreover, LLPS droplets of N_FL_ and N_182-419_ are significantly enlarged in the presence of viral RNAs, and we observed filamentous structures in the N_182-419_ droplets using correlative light and electron microscopy (CLEM), suggesting that the formation of LLPS droplets may promote higher-order assembly of N protein for transcription, replication and packaging. Together this study expands our understanding of the multiple functions of N protein in SARS-CoV-2.

**Supplementary Information:**

The online version contains supplementary material available at 10.1186/s43556-023-00129-z.

## Introduction

Severe acute respiratory syndrome coronavirus 2 (SARS-CoV-2) continues to evolve as it spreads around the world, with the Omicron subvariant XBB currently dominating the global infections[1]. SARS-CoV-2 is a non-segmented positive-stranded RNA virus with strong infectivity and high lethality [2]. The genome of SARS-CoV-2 is almost 30,000 nucleotides (nts), which encodes 16 non-structural proteins (nsp1-nsp16) and 4 structural proteins (Spike, S; Envelope, E; Membrane, M; Nucleocapsid, N) [3–6]. The N protein is located near the 3' end of the genome and is one of the most abundant viral proteins [7]. The N protein assembles into nucleocapsids to wrap and protect the viral genomes. The structural proteins S, E and M form the envelopes of SARS-CoV-2 virus particles that wrap the nucleocapsids [8, 9].

**Fig. 1:**
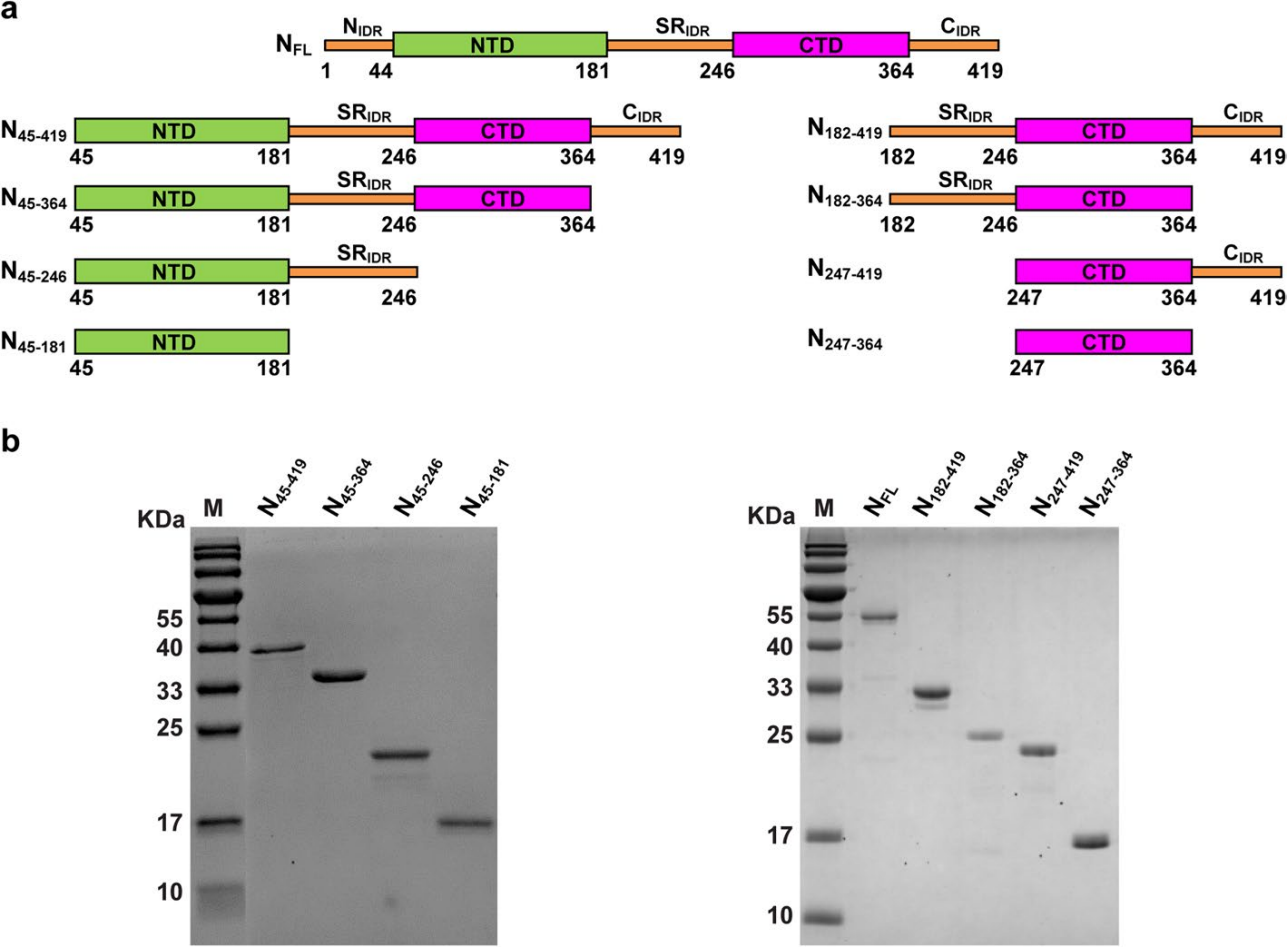
Modular SARS-CoV-2 N constructs. **a** Cartoon illustration of modular N constructs. **b** SDS-PAGE analysis of purified modular N constructs

N is a multifunctional protein that involves in the virus life cycle and cell response to protect the viral genome and regulate the host immune system during virion packaging, as well as participation in the replication and synthesis of genomic RNAs [10–14]. N consists of two structural domains, the N-terminal RNA binding domain (NTD) and the C-terminal dimerization domain (CTD), which are incorporated into three intrinsic disordered regions (IDRs) at the N-terminus of NTD (N_IDR_), between the NTD and CTD with the serine/arginine rich motif (SR_IDR_), and at the C-terminus of CTD (C_IDR_) (Fig. 1a). The sequences of N and the structures of NTD and CTD are highly conserved among SARS-CoV-2 and other β-coronaviruses (CoV), such as SARS-CoV, MERS-CoV and HCoV-OC43 [3, 15]. The NTD has a unique RNA binding pocket, whereas the CTD dimerizes in solution and occasionally forms tetramers in the presence of C_IDR _[16–18]. Small molecules designed to target NTD and CTD binding sites could inhibit RNA binding and oligomerization of N, thereby decreasing the virus replication [15]. N is also highly immunogenic due to its high abundancy and interference with host immune systems during viral infections [19–23], suggesting N as a potential target for diagnosis and biotherapy development.

Filamentous nucleocapsid-like structures in β-CoV including SARS-CoV and SARS-CoV-2 characterized by cryo-electron microscopy (cryo-EM) and cryo-electron tomography (cryo-ET) have been previously reported [24–30], recent cryo-ET analyses of SARS-CoV-2 virions in situ revealed low-resolution globular-shaped ribonucleoprotein (RNP) complexes [31–35]. In the meantime, recent studies on SARS-CoV-2 N protein revealed liquid–liquid phase separation (LLPS) in the presence of SARS-CoV-2 viral RNAs [29, 36–43]. The SR_IDR_ contains a SR-rich motif that may facilitate multimerization of N and transcription-regulating sequence (TRS)-dependent LLPS [44, 45], indicating that LLPS formation could initiate nucleocapsid-like assembly that is essential for transcription, replication and virion packaging.

Here, we present a modular approach to investigate functions of individual domains of SARS-CoV-2 N in protein assembly and LLPS. Recombinant expression and purification of the full-length N protein (N_FL_) from *Escherichia coli* (*E. coli*) revealed ring-like assembly in the presence or absence of cellular RNAs. Intriguingly, the SR_IDR_-CTD-C_IDR_ (N_182-419_) construct with the truncated N_IDR_-NTD showed filamentous assembly. Further truncations of either IDR resulted in ring-like assembly, indicating that both SR_IDR_ and C_IDR_ adjacent to CTD are indispensible for filament formation, whereas N_IDR_-NTD might be inhibitory to filamentous assembly. The higher-order structures, including ring-like and filamentous assembly, were recapitulated in vitro when the N constructs were incubated with previously proposed packaging signals (PS) including 5' UTR (nts 1–478) [46, 47], PS100 (nts 691–789) [48], PS576 (nts 19,786–20,361) [48, 49], PS9 (nts 20,080–21,171 that partially encodes nsp15-16) [50], or PS97 (nts 29,027–29,129) [48, 51] regions of the SARS-CoV-2 genome, whereas 3' UTR could not facilitate protein assembly, validating that these regions likely contain viral assembly and packaging signals. In addition to previous studies that extensively characterized the N_FL_ for LLPS, we further characterized these modular constructs of N for LLPS and observed significantly enlarged droplets containing cylindrical filaments for SR_IDR_-CTD-C_IDR_ in the presence of SARS-CoV-2 5' UTR, suggesting that formation of LLPS droplets may favor the higher-order assembly of N essential for viral RNA synthesis and virion packaging. Together our results provide modular functions of individual domains of N that further complete our understanding of the multi-functional N protein, which may aid novel diagnosis and antiviral development against SARS-CoV-2.

## Results

### Modular characterizations of SARS-CoV-2 N constructs in higher-order assembly with cellular and viral RNAs

**Fig. 2:**
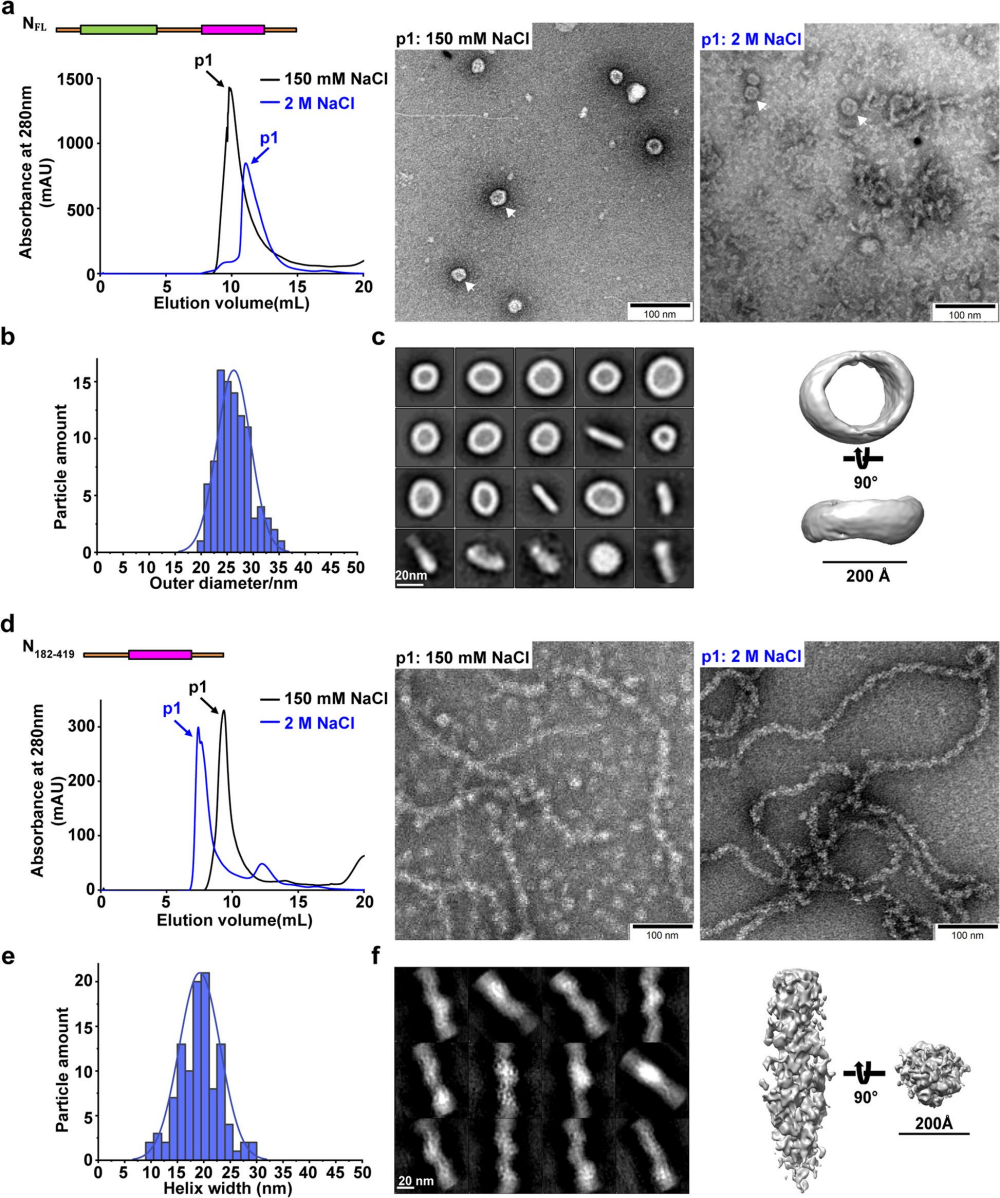
SARS-CoV-2 N_FL_ and N_182-419_ protein assembly under physiological relevant conditions. **a** The PEVs of SARS-CoV-2 N_FL_ protein under physiological relevant salt condition with retained cellular RNAs (black) and high salt condition with removed cellular RNAs (blue) by gel filtration chromatography and the corresponding representative negative stain micrographs. Scale bars, 100 nm. **b** Diameter distribution of N_FL_ protein ring-like particles. **c** 2D averages and 3D reconstruction of N_FL_ negative stain data. Scale bar, 20 nm. **d** The PEVs of SARS-CoV-2 N_182-419_ protein under physiological relevant salt condition with retained cellular RNAs (black) and high salt condition with removed cellular RNAs (blue) by gel filtration chromatography and the corresponding representative negative stain micrographs. Scale bars, 100 nm. **e** Diameter distribution of N_182-419_ filamentous nucleocapsid-like particles. **f** 2D averages and 3D reconstruction of N_182-419_ negative stain data. Scale bar, 20 nm

We designed a modular approach with domain truncations on N-terminus and C-terminus, resulting in eight different modular constructs of N (Fig. 1). Recombinant expression in *E. coli* and purification of the N_FL_ under physiological relevant salt (150 mM NaCl) or high salt (2 M NaCl) conditions resulted in the peak elution volumes (PEVs) of the first peak (p1) as 9.89 mL and 11.06 mL in gel filtration chromatography (Fig. 2a), suggesting possible formations of higher-order assembly. Absorbance measurement showed the existence of cellular nucleic acid under 150 mM NaCl condition, whereas minimal nucleic acid was detected after gel filtration in the presence of 2 M NaCl, which was typically used to remove nonspecifically bound cellular nucleic acids and to induce salt bridge interactions that are frequently observed in protein assembly interfaces [37, 52, 53]. Negative staining revealed heterogeneous ring-like assembly of N under physiological relevant salt condition with the diameter ranging from 20–35 nm (Fig. 2a-b, black p1), which led to the low-resolution three-dimensional (3D) reconstruction of a representative ring-like structure (Fig. 2c).

**Fig. 3:**
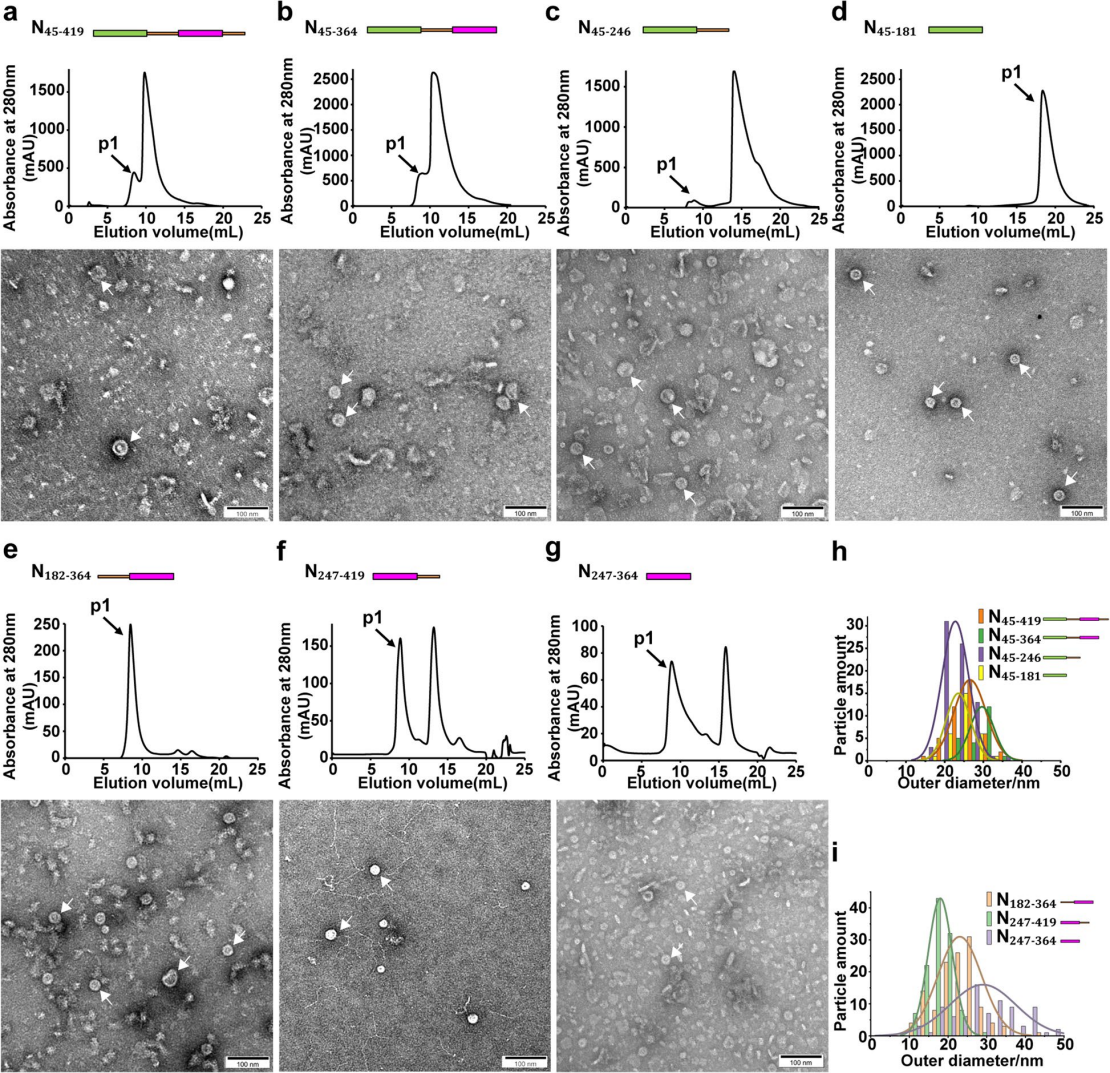
The assembly of different N constructs under 2 M NaCl high salt condition. **a-g** The gel filtration chromatography profiles and representative negative staining images with ring-like structures of (**a**) N_45-419_, (**b**) N_45-364_, (**c**) N_45-246_, (**d**) N_45-181_, (**e**) N_182-364_, (f) N_247-419_ and (g) N_247-364_ under 2 M NaCl condition with cellular RNAs removed. Scale bars, 100 nm. **h-i** Diameter distributions of (**h**) N_45-419_, N_45-364_, N_45-246_, N_45-181_ and (**i**) N_182-364_, N_247-419_, N_247-364_ ring-like assembly showed variable sizes. White arrows indicate ring-like structures

**Fig. 4:**
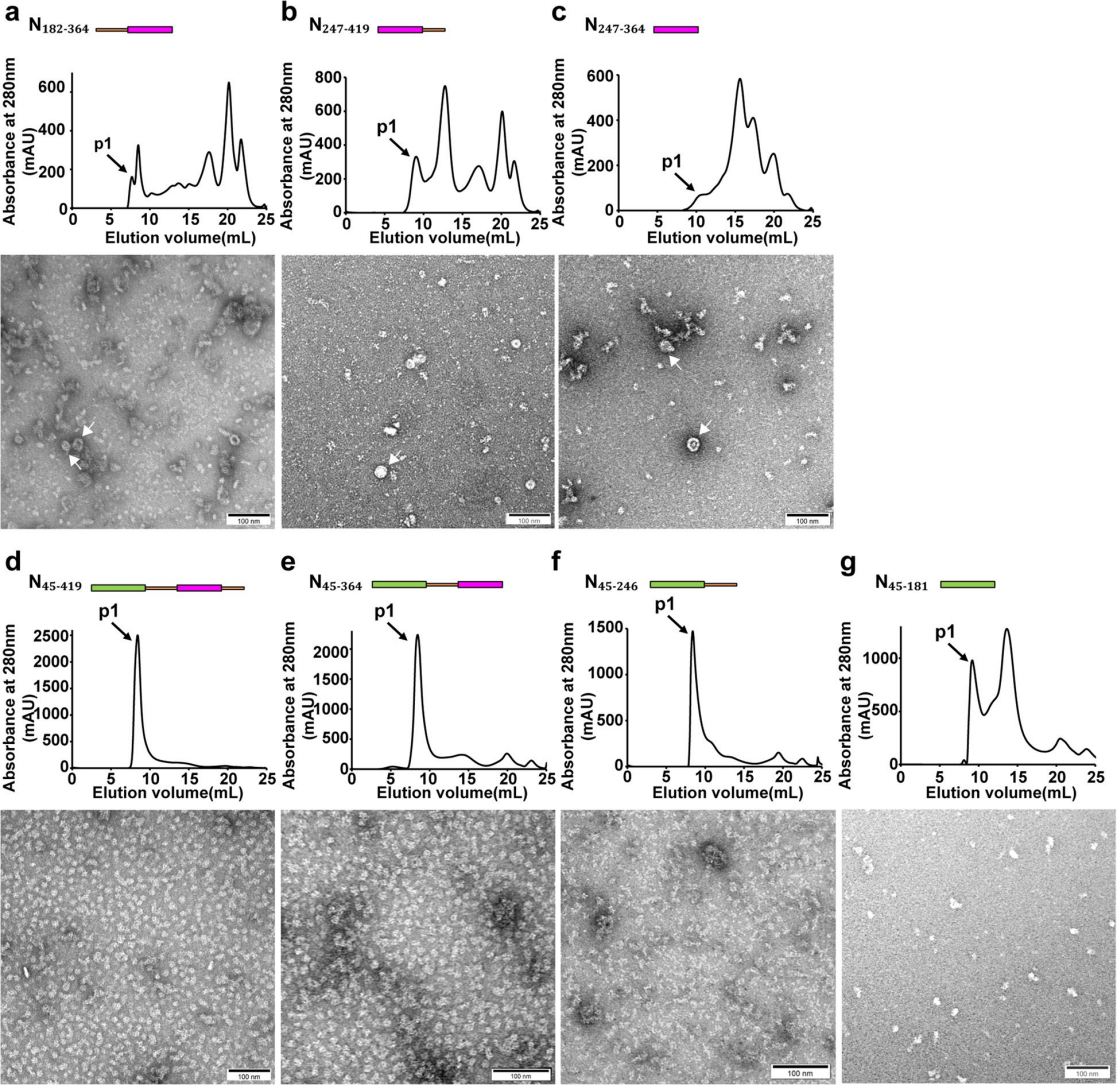
The assembly of different N constructs under 150 mM NaCl physiological relevant salt condition. **a-g** The gel filtration chromatography profiles and representative negative staining images with ring-like structures of (**a**) N_182-364_, (**b**) N_247-419_, (**c**) N_247-364_, and without ring-like structures of (**d**) N_45-419_, (**e**) N_45-364_, (**f**) N_45-246_ and (**g**) N_45-181_ under 150 mM NaCl condition with cellular RNAs. Scale bars, 100 nm. White arrows indicate ring-like structures

We generated eight additional truncated constructs consisted of different modular domains to further characterize their functions in higher-order assembly (Fig. 1). Although all truncated constructs could form higher-order structures to some extent under 2 M NaCl condition (Figs. 2 and 3), indicative of the universal effect of salt on protein assembly [54, 55], only those constructs that contained CTD but not NTD formed higher-order assembly in the presence of RNAs under physiological relevant salt condition (Fig. 4), consistent with previous studies that CTD domain of N was prone to oligomerization [30, 55–57]. The ring-like assembly no longer existed when an additional NTD domain were included, yielding constructs N_45-419_ and N_45-364_, indicating that NTD played an inhibitory role in higher-order N assembly (Fig. 4d-e). The fact that inclusion of N_IDR_ domain in N_FL_ facilitated ring-like assembly suggested the presence of N_IDR_ domain reduced the inhibitory effect of NTD in higher-order N assembly (Fig. 2a).

### N_182-419_ forms filamentous assembly

Previous studies observed filamentous nucleocapsid-like assembly of N in β-CoV including SARS-CoV and SARS-CoV-2 [24–30], whereas recent cryo-ET analyses of SARS-CoV-2 virions in situ revealed low-resolution globular-shaped viral RNPs [31–35], suggesting that the filamentous form of RNP might only exist in the process of RNA synthesis and the beginning of genome packaging to protect the viral genome. Intriguingly, we observed flexible filamentous assembly of N_182-419_ under both high and physiological relevant salt conditions with helix width ranging from 10–30 nm that generally matched the diameter of the previous ring-like assembly (Fig. 2d-e), yielding a low-resolution 3D reconstruction of the representative filament with an averaged diameter of 20 nm (Fig. 2f). The fact that only N_182-419_ construct assembled into filaments suggests that the N_IDR_ and NTD domains might have inhibitory effects, whereas SR_IDR_ and C_IDR_ have displayed augmented effects on filamentous assembly.

### SARS-CoV-2 packaging signals induce filamentous assembly in vitro

**Fig. 5:**
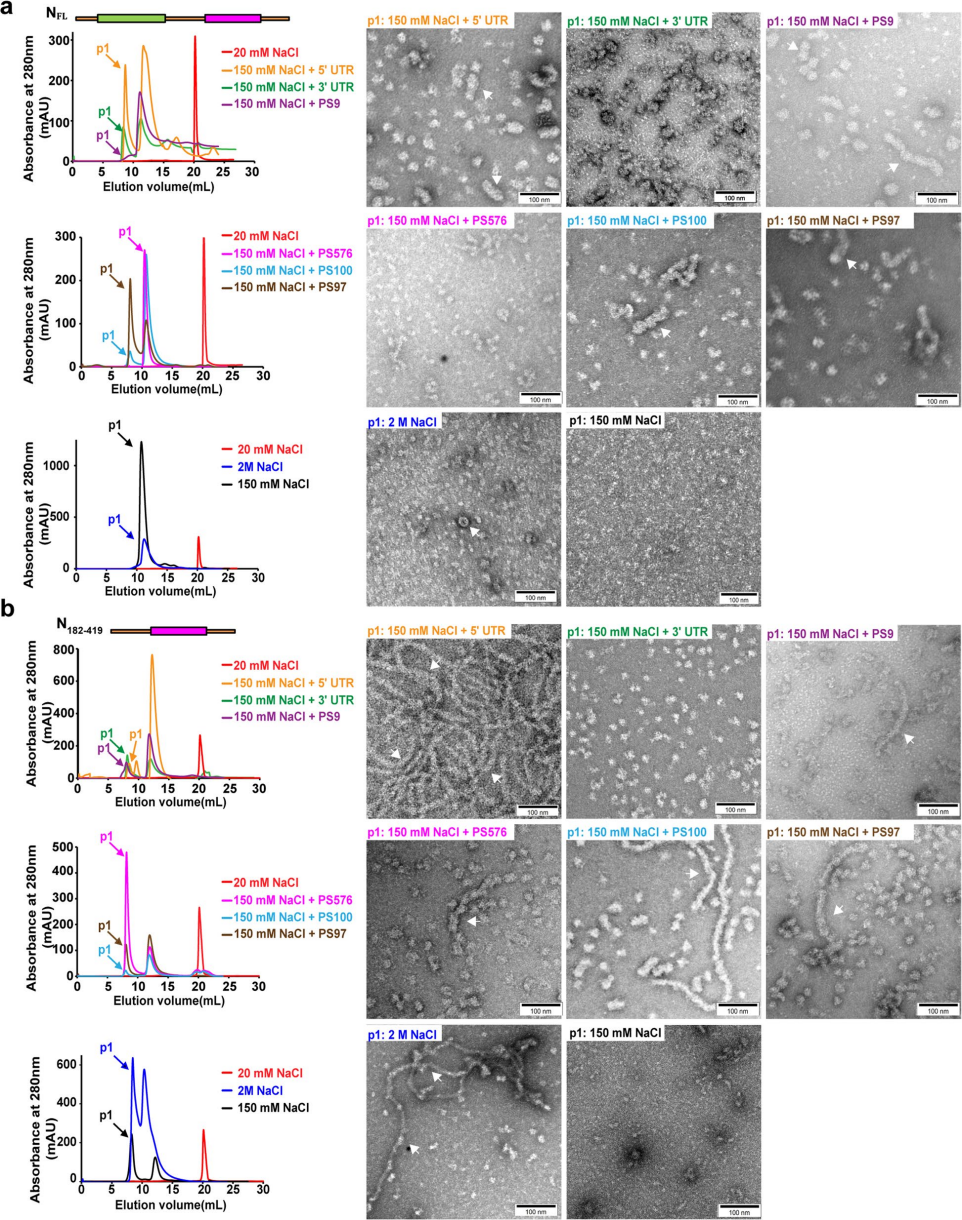
N_FL_ and N_182-419_ assembly in the presence of SARS-CoV-2 RNAs in vitro. **a-b** The dimeric N_FL_ (**a**) and N_182-419_ (**b**) without cellular RNAs were obtained by buffer exchange from 2 M NaCl high salt condition to 20 mM NaCl extremely low salt condition (red), then incubated under 150 mM NaCl physiological relevant salt condition in the presence of SARS-CoV-2 5' UTR (yellow), 3' UTR (green), PS9 (purple), PS576 (magenta), PS100 (cyan), PS97 (brown), 2 M NaCl high salt condition (blue) or 150 mM NaCl physiological relevant salt condition (black) with representative negative staining images under each condition. Scale bars, 100 nm. White arrows indicate filamentous structures

Previous studies demonstrated that the SARS-CoV-2 5' UTR, PS100, PS576, PS9 and PS97 regions on the viral genome could facilitate N protein assembly that are essential for viral ribonucleoprotein formation, virion packaging and viral replication [46, 50, 58]. In order to evaluate the impacts of viral RNAs on N protein assembly, we first obtained the dimeric N_FL_ and N_182-419_ by buffer exchanging from high salt to low salt (20 mM NaCl) to disrupt existing protein assembly (Fig. 5). The absence of nucleic acids was confirmed by absorbance measurement and SDS-PAGE detection (Supplementary Fig. 1). Either 5' UTR, PS100, PS576, PS9, PS97 or 3' UTR of SARS-CoV-2 were folded in the presence of 10 mM Mg^2+^ before incubation with the dimeric N constructs under physiological relevant salt condition (150 mM NaCl) with a ratio of RNA to protein as 1:100, which is determined according to an estimated average ratio of RNA to protein based on previous structural information of N-RNA complexes of single-stranded RNA (ssRNA) viruses [59], since the full-length SARS-CoV-2 N-RNA complex is not resolved. Gel filtration chromatography revealed large shift of the PEVs of different N constructs from ~ 20 mL to prior to 10 mL in the presence of different viral RNAs (Fig. 5). The presence of viral RNAs were confirmed by absorbance measurement. While the N_FL_ could assemble into short filaments, N_182-419_ could assemble back to long filaments in the presence of PS, but not 3' UTR, validating that PS could facilitate N assembly. Electrophoresis mobility shift assay (EMSA) suggested that all PS bind to N_FL_ and N_182-419_ constructs (Supplementary Fig. 1), indicating that higher-order assembly might be facilitated by direct binding of PS to N proteins. We also tested if we could recapitulate the N protein assembly in vitro by incubations of the N constructs under different salt conditions without viral RNAs. Although we have not observed any N assembly under physiological relevant salt condition (150 mM NaCl), under high salt condition (2 M NaCl) the N_FL_ showed some ring-like assembly whereas N_182-419_ showed filamentous assembly as expected, confirming that the N assemblies we observed could reversibly disassemble into dimeric N constructs.

### N and N_182-419_ constructs form variable phase separated droplets that could be enlarged by SARS-CoV-2 5' UTR

**Fig. 6:**
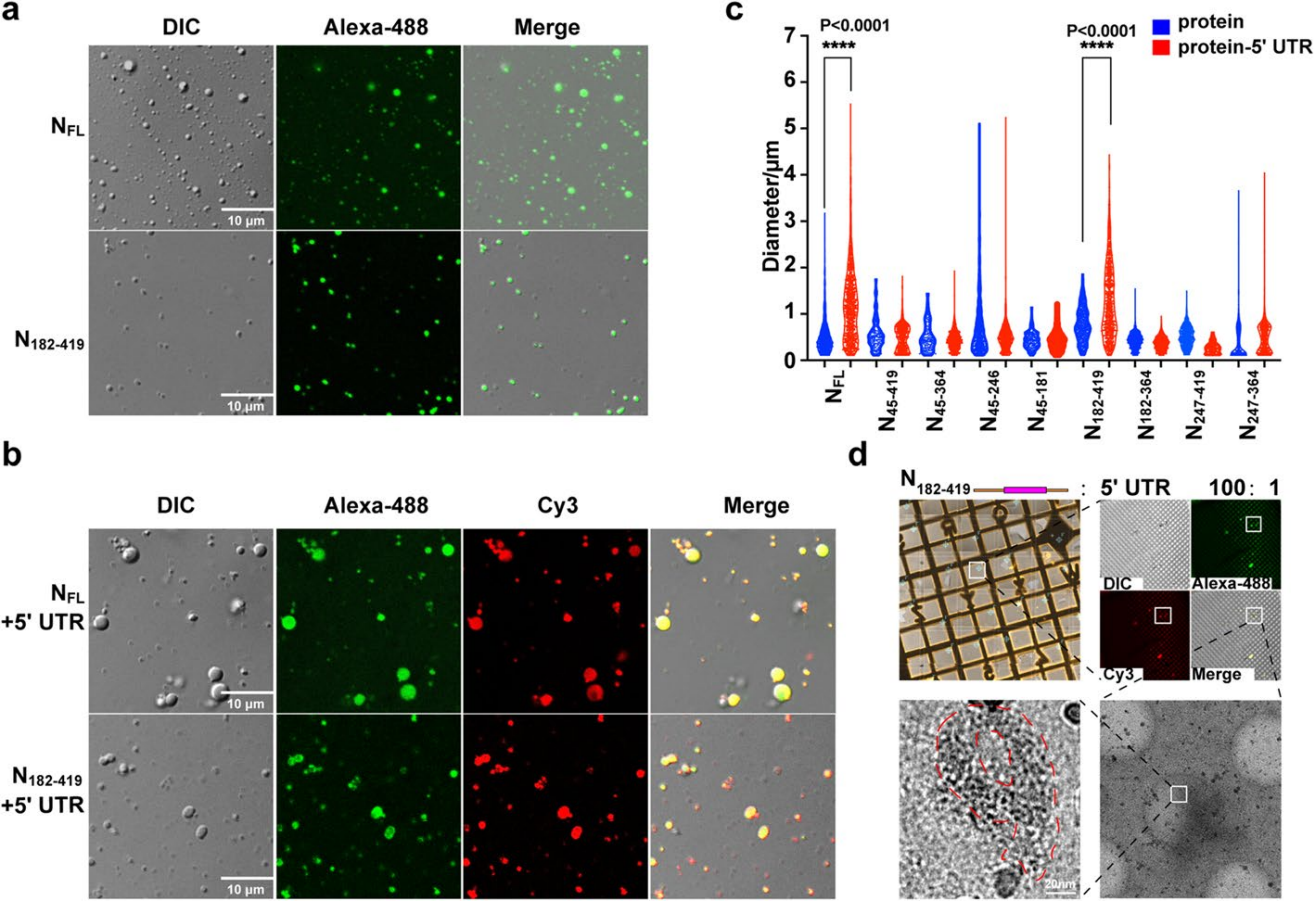
LLPS droplets formed by N_FL_ and N_182-419_ could be enlarged by viral 5' UTR that facilitates protein assembly. **a** N_FL_ and N_182-419_ induced phase separation and formed droplets of variable sizes. Scale bars, 10 μm. **b** Viral 5' UTR enlarged LLPS droplets formed by N_FL_ and N_182-419_. Scale bars, 10 μm. **c** Diameter analysis of ~ 1000 droplets of each construct revealed that droplets formed by N_FL_ and N_182-419_ in the presence of 5' UTR were significantly enlarged. **d** CLEM correlated fluorescent LLPS droplets formed by N_182-419_ under cryo-EM, in which curved filamentous structures were observed (outlined by red dashed line). Scale bar, 20 nm. ****P < 0.0001 by two-tailed Student’s T-test

SARS-CoV-2 N protein undergoes LLPS to form partially ordered gel-like condensates for RNA compaction that could potentially facilitate viral RNA synthesis and genome packaging [29, 36–39, 41]. However, modular functions of individual domains of N in LLPS and the impacts of LLPS on N assembly remain underexplored. We found that all N constructs formed droplets of similar diameters in the presence or absence of 5' UTR (Fig. 6, Supplementary Fig. 2), except that the droplets formed by N_FL_ and N_182-419_ were significantly enlarged by adding 5' UTR RNA (Fig. 6a-c). We used correlative light and electron microscopy (CLEM) to correlate fluorescent droplets of N_182-419_ and 5' UTR under cryo-electron microscopy (cryo-EM), in which we observed curved filamentous structures of roughly 20 nm diameter (Fig. 6d). This result suggests that the compact SARS-CoV-2 RNA-N protein condensates formed by LLPS could be enlarged in order to facilitate N assembly for viral RNA synthesis and packaging.

## Discussion

Many ssRNA viral nucleocapsids that form filamentous assembly were found to pack as ring-like assembly using X-ray crystallography or cryo-EM [60–62]. SARS-CoV-2 N protein is highly conserved and abundant, and previous studies also suggested that N might assemble into filaments to protect viral RNA during transcription, replication, and packaging [24–30].

In this study, we first observed formation of ring-like structures in N_FL_ that suggest helical assembly of the nucleocapsid in ssRNA viruses [60, 63]. Subsequently, we generated multiple modular constructs of SARS-CoV-2 N protein and uncovered the impact that individual domain has on protein assembly. Intriguingly, N_182-419_ that contains SR_IDR_-CTD-C_IDR_ domains could assemble into filaments that may play essential roles as templates for viral transcriptions and replications: The SR_IDR_ domain could drive LLPS to suppress host immune system that is critical for viral genome packaging [41], and undergo phosphorylation that is critical for transcriptions [64]; The CTD domain could bind to RNA and oligomerize during packaging [30], and bind to nsp3 to play a crucial role in transcriptions [65]; The function of C_IDR_ domain is associated with M that also affects viral packaging [41].

Several segments on the viral genomes of SARS-CoV and SARS-CoV-2 have been previously suggested as the packaging signals, including 5' UTR, PS9, PS69, PS97, PS100 and PS576 [41, 47–49, 48]. Intriguigingly, the filament could be assembled in the presence of PS, whereas addition of 3' UTR viral RNA would not result in filament formation. Multiple studies have shown that mutations and repetitive structural motifs in 5' UTR were critical for viral packaging in various coronaviruses [46, 47, 66–69], whereas PS9 has been recently identified as the minimal packaging signal element for SARS-CoV-2 virus-like particle [50].

All constructs that contained CTD formed either ring-like or filamentous higher-order assembly that could be disrupted by inclusion of the NTD domain in the constructs under the physiological relevant salt condition that preserved cellular RNAs, which suggested that NTD could suppress higher-order N assembly. The fact that N_FL_ could still form ring-like structures indicated that inclusion of the N_IDR_ domain in N_FL_ could reduce the inhibitory effect of NTD against higher-order N assembly. Previous study has reported that the N-terminus, N_1-209_, could bind to the host factor cyclophilin A, which participates in replication cycle of coronaviruses and other viral assembly processes [70]. Since numerous proteins from host cells have been suggested to interact with N [71, 72], it is possible that during viral transcriptions and replications in cells, additional viral and host factors may interact with NTD to completely diminish its inhibitory role in higher-order N assembly so that filamentous nucleocapsids could be formed as templates for efficient viral transcription and replication. Future study of N assembly formations in human cells or cell lysates may shed light on the N-terminal functions.

**Fig. 7:**
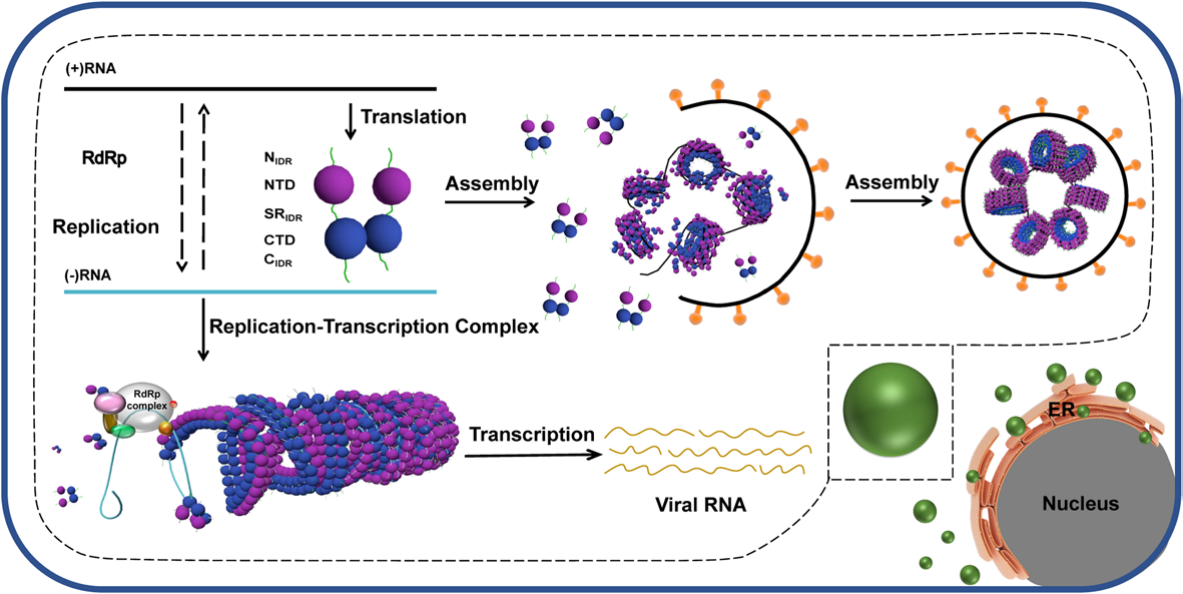
SARS-CoV-2 N protein assembly facilitated by phase separation. SARS-CoV-2 N protein participates in viral RNA transcription and replication, and forms nucleocapsid-like assembly to protect the viral RNA template during transcription. N protein also initiates viral genomic RNA packaging that eventually forms multiple globular ribonucleoprotein complexes. LLPS induced by N protein might enable the assembly processes

LLPS has been suggested to condense viral proteins and RNAs for efficient packaging and replication [73–76]. SARS-COV-2 N protein has been previously reported to undergo LLPS [38]. Intriguingly, we noticed that addition of viral 5' UTR to both N_FL_ and N_182-419_ enlarged the droplet size with flexible filamentous structures observed in N_182-419_ droplets under CLEM, indicating that larger space might facilitate higher-order N assembly. We propose a working model of N that undergoes LLPS to condensate with SARS-CoV-2 genomic RNA to facilitate RNP assembly (Fig. 7).

In conclusion, we have carried out a modular approach to characterize functions of individual domains of SARS-CoV-2 N in protein assembly and LLPS. These findings further complete our understandings in N protein assembly that may play essential role in RNA replication and packaging.

## Materials and methods

### Modular construct design and plasmid construction

The pET28a-SARS-CoV-2-N_FL_ plasmid was kindly provided by Guangdong Laboratory Animal Testing Institute. To investigate the function of each domain of SARS-CoV-2 N in protein assembly and LLPS, a modular research approach was designed based on the feature that the N protein consists of two domains and three intrinsically disordered regions. Centering on the two domains, we truncated the intrinsic disordered regions at the N-terminus and C-terminus step by step, and constructed eight different N-modular mutants. The SARS-CoV-2-N truncations (pET-28a-N_45-419_, pET-28a-N_45-364_, pET-28a-N_45-246_, pET-28a-N_45-181_, pET-28a-N_182-419_, pET-28a-N_182-364_, pET-28a-N_247-419_, pET-28a-N_247-364_) were amplified with pET28a-SARS-CoV-2-N_FL_ as the template and cloned into the pET-28a vector between NdeI and XhoI by ClonExpress® II one step cloning kit (Vazyme). The 5' UTR, PS100, PS576, PS9, PS97 or 3' UTR were designed by combining a T7 RNA polymerase promoter sequence and the hepatitis δ virus ribozyme (HDV) sequence to produce uniform 5' ends, in addition to the desired sequence from the full-length SARS-CoV-2 cDNA, which was a kind gift from Beijing Institute of Microbiology and Epidemiology. The combined sequences were inserted into the pUC-19 vector between HindIII and NdeI restriction sites for RNA transcription and purification.

### Protein expression and purification

pET28a-SARS-CoV-2-N_FL_ and the truncated protein plasmids (Supplementary Table 1) were individually transformed into *Escherichia coli* (*E. coli*) BL21 (DE3) strain (Novagen). *E. coli* cells were cultured in Luria–Bertani (LB) medium at 37 ℃ with 50 mg/L kanamycin until the OD600 reached 0.6–0.8, then the bacteria were induced with 0.5 mM Isopropyl β-D-1-thiogalactopyranoside (IPTG) at 18 ℃ for 15–18 h. Bacteria were collected by centrifugation, resuspended in buffer containing 20 mM Tris–HCl pH 8.0, 150 mM NaCl, 10 mM Imidazole, 5% Glycerol, 1 mM phenylmethylsulfonyl fluoride to retain the bacterial nucleic acids and lysed by Ultrasonic Cell Crushe. Cell extracts were centrifuged at 15,000 × g for 40 min at 4 ℃. Supernatants were purified with Ni–NTA (GE Healthcare), the target protein was washed with lysis buffer and then eluted with a buffer containing 20 mM Tris–HCl pH 8.0, 150 mM NaCl, 300 mM imidazole. Eluted proteins were concentrated by centrifugal ultrafiltration, loaded onto a pre-equilibrated Superdex™ 200 Increase 10/300 GL column in an ÄKTA-purifier (GE Healthcare), eluted at a flow rate of 0.4 ml/min with the same buffer containing 20 mM Tris–HCl pH 8.0, 150 mM NaCl. Peak fractions were analyzed by SDS-PAGE (15%, w/v) and stained with Coomassie brilliant blue R-250. The peak fractions of proteins retaining the bacterial nucleic acids were concentrated to 0.5 mg/ml for negative stain EM. Bacterial nucleic acids were removed under 2 M NaCl condition with the rest of the procedure unchanged in the abovementioned protein purification process.

The N protein assembly was disrupted by buffer exchange from 2 M NaCl high salt condition to 20 mM NaCl low salt condition as confirmed by gel filtration chromatography and SDS-PAGE analysis. Fractions were pooled together and concentrated by centrifugal ultrafiltration (Millipore). All sample concentrations were determined by A280 (NanoDrop One^C^, Thermo Scientific). The storage protein samples were quick-frozen by liquid nitrogen then kept at-80 ℃ before use.

### RNA preparation

The SARS-CoV-2 5' UTR, PS9, PS576, PS100, PS97 and 3' UTR DNA templates in pUC-19 plasmid (Supplementary Table 1) were amplified by Qiagen MegaPrep kits. Linear dsDNA templates were acquired with the regular forward primer and 2'-O-methylated reverse primer to improve 3' end homogeneity by PCR [77] (See Supplementary Table 2 for primers). The resulting DNA templates were isolated by ethanol precipitation. In vitro transcription of SARS-CoV-2 RNAs were carried out with 1.5–2 µg DNA templates, 4 mM NTPs and 1 U/µL RNase inhibitor in 1 × transcription buffer containing 40 mM Tris–HCl (pH 7.9), 0.01% TritonX-100, 20 mM MgCl_2_, 2 mM spermidine, 10 mM DTT, and incubated at 37 ℃ for 3 h. The transcription products were mixed with 2 × denaturing gel loading buffer containing 95% formamide, 0.025% SDS, 10 mM EDTA, 0.025% xylene cyanol, and 0.025% bromophenol blue, and loaded on an 6–10% 29:1 acrylamide:bis, 7 M urea polyacrylamide gel. The gel was run at 300 V for 6 h, then stained for 5 min in SyBrGold (Invitrogen) and visualized by UV transillumination Molecular Imager (Biorad ChemiDoc™ XRS +). RNA ladder was purchased in Thermo Scientific (SM1833). To acquire SARS-CoV-2 RNAs for assembly with N protein in vitro, RNAs were gel purified and visualized briefly with a 254-nm UV lamp, held far from the gel to minimize RNA damages [78]. Then the RNAs were eluted from the gel overnight in elution buffer containing 30 mM sodium acetate (pH 5.5) and 1 mM EDTA on an active vortexer at 4 ℃ overnight. The resulting gel slurry was then filtered through 0.45 μm filters (Minisart® Syringe Filter, Sartorius). The resulting RNAs were precipitated with isopropyl alcohol to remove urea and salts. The products were pelleted by centrifugation at 15,000 × g for 1 h at 4 ℃, washed with 80% cold ethanol for three times and dried in a Speedvac, then resuspend the pellet in RNase-free water. RNA was quantified using a NanoDrop One^C^ (Thermo Scientific) and kept at -80 ℃ before use.

### RNA refolding

The 5' UTR, PS9, PS576, PS100, PS97 and 3' UTR RNAs were added to refolding buffer (10 mM Tris–HCl pH 7.4, 100 mM KCl). The RNA solution was heated at 90 ℃ for 3 min, cooled to 25 ℃ for 10 min, then added MgCl_2_ to a final concentration of 10 mM, followed by incubation at 50 ℃ for 30 min and 25 ℃ for 15 min. Refolded RNA samples were mixed with 5 × gel loading buffer containing 10% glycerol, 0.025% xylene cyanol, and 0.025% bromophenol blue, and loaded on an 6% 29:1 acrylamide: bis polyacrylamide gel. The gel was run at 110 V for 1.5 h, then stained for 5 min in SyBrGold (Invitrogen) and visualized by UV transillumination Molecular Imager (Biorad ChemiDoc™ XRS +). The refolded RNAs were kept on ice before use.

### N protein assembly in vitro

All dimeric N constructs were incubated with refolded viral 5' UTR, PS9, PS576, PS100, PS97 or 3' UTR with a protein to RNA molar ratio of 100:1, at 37 ℃ for 30 min in the assembly buffer containing 20 mM Tris–HCl pH 8.0, 10 mM KCl, 140 mM NaCl, 1 mM MgCl_2_. In the absence of viral RNAs, dimeric N constructs were incubated in buffer containing 20 mM Tris–HCl pH 8.0, 2 M NaCl to obtain assembly under high salt condition. The resulting assembly mixtures were loaded onto a pre-equilibrated Superdex™ 200 Increase 10/300 GL column in an ÄKTA-purifier (GE Healthcare), eluted at a flow rate of 0.4 ml/min with the buffer containing 20 mM Tris–HCl pH 8.0, 10 mM KCl, 140 mM NaCl, 1 mM MgCl_2_ in the presence of viral RNAs, and 20 mM Tris–HCl pH 8.0, 2 M NaCl in the absence of viral RNAs. Peak fractions were analyzed by SDS-PAGE (15%, w/v) and stained with Coomassie brilliant blue R-250. All fractions were concentrated to 0.5 mg/ml for negative stain EM.

### EMSA assay

The 9 pmol refolded SARS-CoV-2 5' UTR, PS9, PS576, PS100 or PS97 binds to N_FL_ protein or N_182-419_ (storage buffer: 20 mM Tris–HCl pH 8.0, 20 mM NaCl) by the ratio of 1:1, 1:2, 1:4, 1:8 in buffer 10 mM Tris–HCl pH 7.4, 10 mM NaCl, 1 mM KCl, 10 mM MgCl_2_. After incubating at 37 ℃ for 30 min, SARS-CoV-2 5' UTR, PS576, PS100 or PS97 detect with 6% native urea gel and PS9 1% native agarose gel (1000nts RNA for native urea gel is too long, 6% native urea gel can’t be detected, but data not show), then stained for 5 min in SyBrGold (Invitrogen) and visualized by UV transillumination Molecular Imager (Biorad ChemiDoc™ XRS +).

### Fluorescence labeling and LLPS assay

The dimeric N constructs with removed cellular nucleic acids were labeled by fluorescence dye Oregon-Green488 (Invitrogen, 2,161,802) with a molar ratio 10:1 between protein and fluorescence dye in labeling buffer 50 mM NaPhosphate pH 7.0, 50 mM NaCl. The mixtures were incubated at 25 ℃ for 1 h. To remove the excess fluorescence dye, the resulting mixture was buffer-exchanged with labeling buffer using concentrator columns with 10 kDa cutoff (Ultrafiltration Centrifugal Tube, Millipore). The SARS-CoV-2 5' UTR and 3' UTR were labeled by fluorescence dye Cy3 (Lumiprobe, #41,070) following the Cy3 labelling protocol [79]. The labeled proteins and RNAs were quantified using a NanoDrop One^C^ (Thermo Scientific).

Oregon-Green488 labeled N constructs were diluted to a final concentration of 25 μM in phase separation buffer (50 mM Tris–HCl pH 7.5, 100 mM NaCl) containing 10% PEG 3350, and RNAs were added with a protein to RNA molar ratio of 100:1. Then the mixture was incubated at room temperature for 5 min. A total of 10 μL solution was transferred onto the glass slide, and images were collected using a Zeiss-Axio Observer 7 microscope.

### CLEM imaging

A total of 3 μL of the LLPS sample containing N_182-419_ and 5' UTR was applied onto glow-discharged (45 s) Quantifoil Au H2 finder (R 2/1) grids (Quantifoil Micro Tools GmbH, Germany). The grids were blotted with filter paper for 2.5 s in 100% humidity at 4 ℃ with no blotting offset and rapidly frozen in liquid ethane using a Vitrobot Mark IV (Thermo Fisher). The fluorescent images were collected by LEICA EM Cryo CLEM microscope and LAS X software at 60 × magnification.

Frozen grids were then loaded into a Titan Krios cryo-electron microscope (Thermo Fisher) operated at 300 kV with a 50 μm condenser lens aperture and spot size 5. Micrographs were collected using EPU software (Version 2.9.0.1519REL) under various magnifications.

### Negative stain EM sample preparation and data collection

Three drops of 20 μL, 20 μL, and 60 μL 0.75% uranium formate (UF) stain solution were applied on the parafilm (Bemis). Then, 3 μL of sample was applied on the glow-discharged (40 s) 300-mesh-Cu grids (Quantifoil) coated with a continuous carbon film and waited 60 s to allow sample adsorption. The grid was blotted from the side with a piece of filter paper and washed with 4 μL of washing buffer. Excessive washing buffer was blotted with filter paper and the grid was stained in the first two drops of UF followed by blotting with filter paper. Subsequently, the grid was stained in the third drop of UF for 40 s. Excessive UF was blotted and the grid was air-dried and stored until imaging. The grid was placed on a side-entry holder and loaded into a JEM-1400 operated at 120 kV, condenser lens aperture 150 μm, spot size 1. Micrographs were collected using RADIUS software on a Morada G3 direct electron camera under magnification of 120,000 × (corresponding to a calibrated sampling of 3.23 Å per physical pixel).

### Image processing and 3D reconstruction

The 99 negative stain images of N_FL_ assembly were subjected to EMAN2 [80] for neural network particle picking. A total of 6,152 particles were extracted in Relion 3.1 [81] with a box size of 140 pixels. After 2D classifications in cryoSPARC3.1 [82], 2,384 particles were subjected to one round of ab-initio reconstruction and heterogeneous refinement to remove poor-quality particles. The best class was selected for homogeneous refinement, and the map after homogeneous refinement was displayed in UCSF Chimera [83].

The 21 negative stain images of N_182-419_ assembly were processed until 2D classification following the same protocol above with a box size of 320 pixels. After 2D classification, 14,761 particles were subjected to helical reconstruction in cryoSPARC3.1 to obtain a low-resolution 3D density map and displayed in UCSF Chimera.

### Statistics and reproducibility

All the experiments were independently repeated for at least three times, and no inconsistent results were observed. Origin software and GraphPad Prism v.9.0.0 was used to perform statistical analyses. To determine the partition coefficient of indicated groups, 3 microscopy images were randomly selected, and the diameter of particles or droplets was acquired with Image J. Data are presented as the mean ± S.D or ± S.E.M. The box borders in the boxplots and violin plots represent the upper and lower quartiles (25th and 75th percentiles), and the center line denotes the median. A standard two-tailed unpaired Student’s t-test was used for statistical analysis of two groups; *p < 0.05, **p < 0.01, ****p < 0.0001, and *p* > 0.05 is considered as non-significant. All the data were reproducible, and details of replicates are described in the figure legends.

## Supplementary Information

**Additional file 1:** **Supplementary Fig 1.** EMSA analysis of N_FL_ or N_182-419_ binding with SARS-CoV-2 5' UTR, PS9, PS576, PS100 and PS97. SARS-CoV-2 5' UTR, PS9, PS576, PS100 or PS97 (9 pmol) and N_FL_ protein or N_182-419_ were combined at a ratio of 1:1, 1:2, 1:4, 1:8. After incubation at 37°C for 30 min, detect with 6% or 1% Native PAGE gel. **Supplementary Fig 2.** LLPS analysis of different N constructs in the presence and absence of viral 5' UTR. a-b N_45-419_, N_45-364_, N_45-246_, N_45-181_, N_182-364_, N_247-419_ and N_247-364_ undergo phase separation to form variable droplets (a) without SARS-CoV-2 5' UTR and (b) with 5' UTR colocalized in the droplets. Scale bars, 10 µm. **Supplementary Table 1.** RNA sequences used in this study. **Supplementary Table 2.** PCR primers used in this paper.

## Data Availability

The EM reconstructions have been deposited in EMDB under EMD accession codes of EMD-35527 for N_FL_ and EMD-35528 for N_182-419_, respectively. All other data are available from the authors upon reasonable request.
