# Supplementary material for: Modular characterization of SARS-CoV-2 nucleocapsid protein domain functions in nucleocapsid-like assembly
Source: Mol Biomed. 2023 May 22;4:16. doi: 10.1186/s43556-023-00129-z (PMC10200704; doi:10.1186/s43556-023-00129-z)
Supplement: Supplementary file 1 — Additional file 1: Supplementary Fig 1. EMSA analysis of NFL or N182-419 binding with SARS-CoV-2 5' UTR, PS9, PS576, PS100 and PS97. SARS-CoV-2 5' UTR, PS9, PS576, PS100 or PS97 (9 pmol) and NFL protein or N182-419 were combined at a ratio of 1:1, 1:2, 1:4, 1:8. After incubation at 37°C for 30 min, detect with 6% or 1% Native PAGE gel. Supplementary Fig 2. LLPS analysis of different N constructs in the presence and absence of viral 5' UTR. a-b N45-419, N45-364, N45-246, N45-181, N182-364, N247-419 and N247-364 undergo phase separation to form variable droplets (a) without SARS-CoV-2 5' UTR and (b) with 5' UTR colocalized in the droplets. Scale bars, 10 µm. Supplementary Table 1. RNA sequences used in this study. Supplementary Table 2. PCR primers used in this paper. [file 43556_2023_129_MOESM1_ESM.docx]

**Supplementary Information**

**Modular characterization of SARS-CoV-2 nucleocapsid protein function in nucleocapsid-like assembly**

Yan Wang^1,§^, Xiaobin Ling^1,2§^, Chong Zhang^1^, Jian Zou^1^, Bingnan Luo^1^, Yongbo Luo^1^, Xinyu Jia^1^, Guowen Jia^1^, Minghua Zhang^3^, Junchao Hu^1^, Ting Liu^1^, Yuanfeiyi Wang^1^, Kefeng Lu^1^, Dan Li^4^, Jinbiao Ma^2,*^, Cong Liu^5,6,*^ and Zhaoming Su^1,*^

^1^*The State Key Laboratory of Biotherapy, Frontiers Medical Center of Tianfu Jincheng Laboratory, National Clinical Research Center for Geriatrics and Department of Geriatrics, West China Hospital, Sichuan University, Chengdu, Sichuan 610044, China.*

^2^*State Key Laboratory of Genetic Engineering, Collaborative Innovation Center of Genetics and Development, Department of Biochemistry, School of Life Sciences, Fudan University, Shanghai 200438, China.*

^3^*College of Polymer Science and Engineering, Sichuan University, Chengdu, Sichuan 610065, China.*

^4^*Bio-X Institutes, Key Laboratory for the Genetics of Developmental and Neuropsychiatric Disorders, Ministry of Education, Shanghai Jiao Tong University, Shanghai 200030, China*

^5^*Interdisciplinary Research Center on Biology and Chemistry, Shanghai Institute of Organic Chemistry, Chinese Academy of Sciences, Shanghai, 201210, China*

^6^*State Key Laboratory of Bio-Organic and Natural Products Chemistry, Shanghai Institute of Organic Chemistry, Chinese Academy of Sciences, Shanghai, 200032, China.*

^§^*These authors contributed equally*

**Correspondence should be addressed to Z.S. (zsu@scu.edu.cn), C.L. (liulab@sioc.ac.cn) and J.M (majb@fudan.edu.cn)*


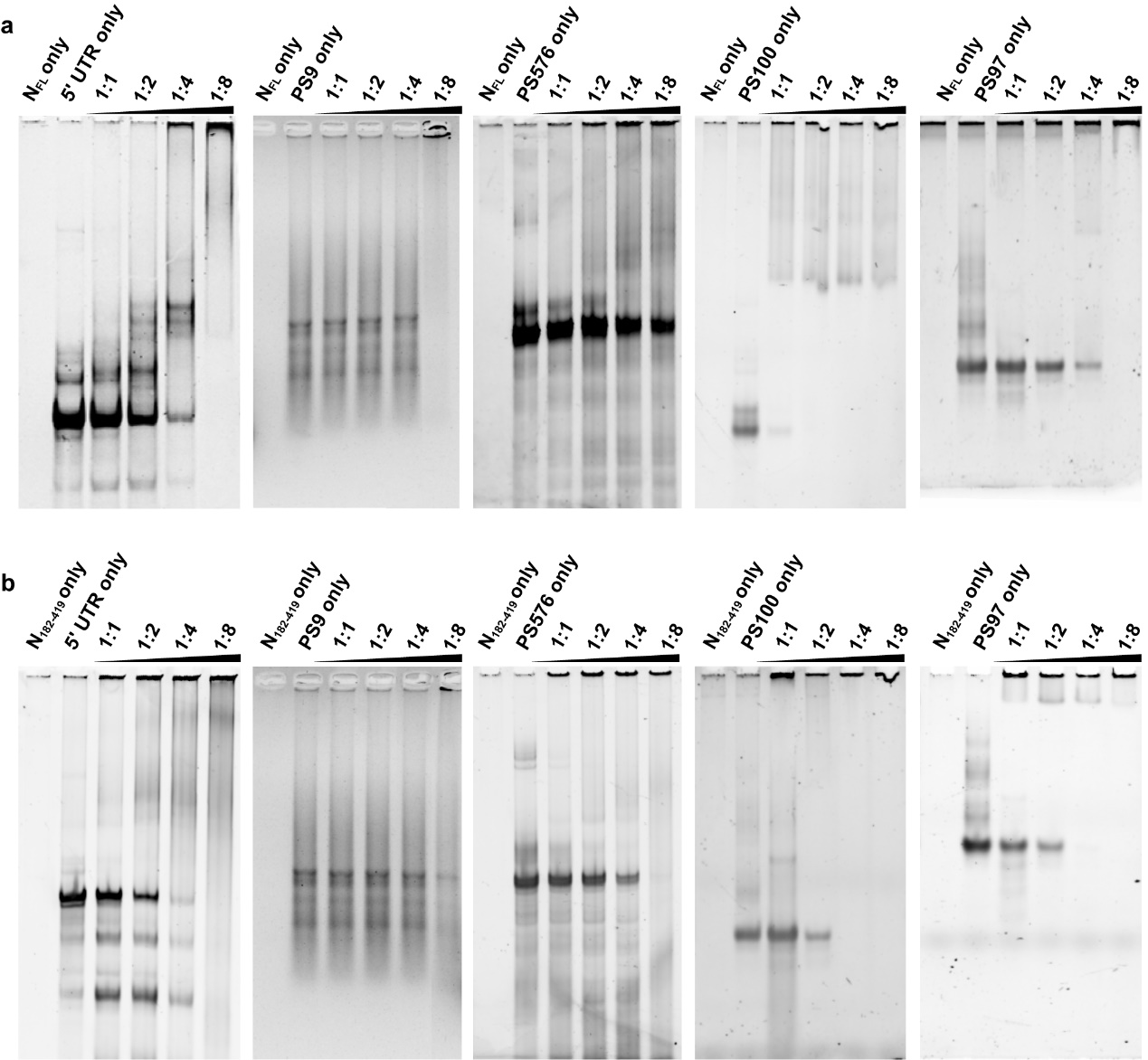


**Supplementary Fig 1: EMSA** analysis of N_FL_ or N_182-419_ binding with SARS-CoV-2 5' UTR, PS9, PS576, PS100 and PS97. SARS-CoV-2 5' UTR, PS9, PS576, PS100 or PS97 (9 pmol) and N_FL_ protein or N_182-419_ were combined at a ratio of 1:1, 1:2, 1:4, 1:8. After incubation at 37°C for 30 min, detect with 6% or 1% Native PAGE gel.


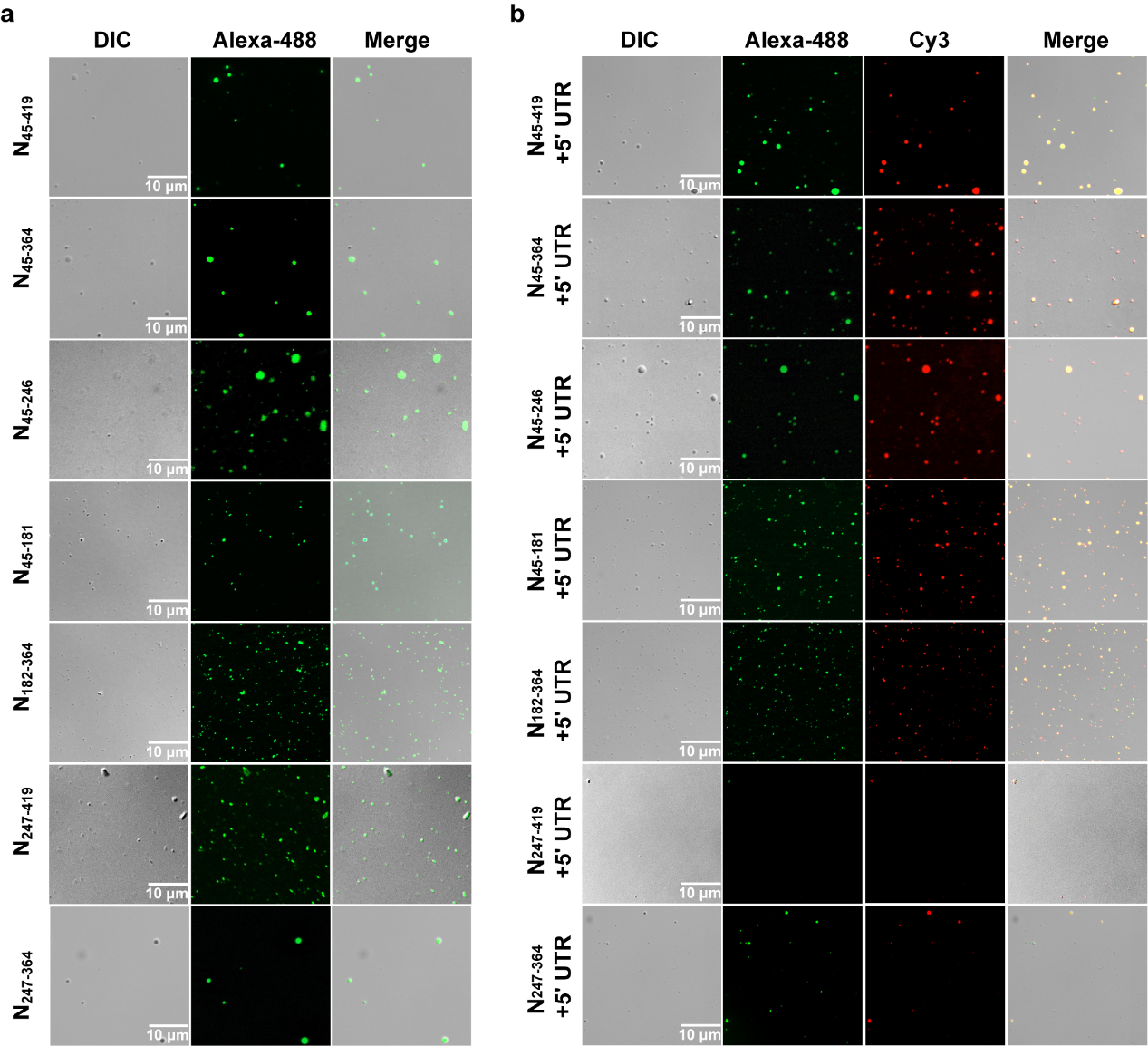


**Supplementary Fig 2: LLPS analysis of different N constructs in the presence and absence of viral 5**' **UTR. a-b** N_45-419_, N_45-364_, N_45-246_, N_45-181_, N_182-364_, N_247-419_ and N_247-364_ undergo phase separation to form variable droplets **(a)** without SARS-CoV-2 5' UTR and **(b)** with 5' UTR colocalized in the droplets. Scale bars, 10 µm.

**Supplementary Table 1: RNA sequences used in this study**

| **Name** | **Sequence (5'-3')** |
| --- | --- |
| **5' UTR** | AUUAAAGGUUUAUACCUUCCCAGGUAACAAACCAACCAACUUUCGAUCUCUUGUAGAUCUGUUCUCUAAACGAACUUUAAAAUCUGUGUGGCUGUCACUCGGCUGCAUGCUUAGUGCACUCACGCAGUAUAAUUAAUAACUAAUUACUGUCGUUGACAGGACACGAGUAACUCGUCUAUCUUCUGCAGGCUGCUUACGGUUUCGUCCGUGUUGCAGCCGAUCAUCAGCACAUCUAGGUUUCGUCCGGGUGUGACCGAAAGGUAAGAUGGAGAGCCUUGUCCCUGGUUUCAACGAGAAAACACACGUCCAACUCAGUUUGCCUGUUUUACAGGUUCGCGACGUGCUCGUACGUGGCUUUGGAGACUCCGUGGAGGAGGUCUUAUCAGAGGCACGUCAACAUCUUAAAGAUGGCACUUGUGGCUUAGUAGAAGUUGAAAAAGGCGUUUUGCCUCAACUUGAACAGCCCUAUGUGUUCAUC |
| **3' UTR** | ACUCAUGCAGACCACACAAGGCAGAUGGGCUAUAUAAACGUUUUCGCUUUUCCGUUUACGAUAUAUAGUCUACUCUUGUGCAGAAUGAAUUCUCGUAACUACAUAGCACAAGUAGAUGUAGUUAACUUUAAUCUCACAUAGCAAUCUUUAAUCAGUGUGUAACAUUAGGGAGGACUUGAAAGAGCCACCACAUUUUCACCGAGGCCACGCGGAGUACGAUCGAGUGUACAGUGAACAAUGCUAGGGAGAGCUGCCUAUAUGGAAGAGCCCUAAUGUGUAAAAUUAAUUUUAGUAGUGCUAUCCCCAUGUGAUUUUAAUAGCUUCUUAGGAGAAUGAC |
| **PS9** | UCUGUAGGUCCCAAACAAGCUAGUCUUAAUGGAGUCACAUUAAUUGGAGAAGCCGUAAAAACACAGUUCAAUUAUUAUAAGAAAGUUGAUGGUGUUGUCCAACAAUUACCUGAAACUUACUUUACUCAGAGUAGAAAUUUACAAGAAUUUAAACCCAGGAGUCAAAUGGAAAUUGAUUUCUUAGAAUUAGCUAUGGAUGAAUUCAUUGAACGGUAUAAAUUAGAAGGCUAUGCCUUCGAACAUAUCGUUUAUGGAGAUUUUAGUCAUAGUCAGUUAGGUGGUUUACAUCUACUGAUUGGACUAGCUAAACGUUUUAAGGAAUCACCUUUUGAAUUAGAAGAUUUUAUUCCUAUGGACAGUACAGUUAAAAACUAUUUCAUAACAGAUGCGCAAACAGGUUCAUCUAAGUGUGUGUGUUCUGUUAUUGAUUUAUUACUUGAUGAUUUUGUUGAAAUAAUAAAAUCCCAAGAUUUAUCUGUAGUUUCUAAGGUUGUCAAAGUGACUAUUGACUAUACAGAAAUUUCAUUUAUGCUUUGGUGUAAAGAUGGCCAUGUAGAAACAUUUUACCCAAAAUUACAAUCUAGUCAAGCGUGGCAACCGGGUGUUGCUAUGCCUAAUCUUUACAAAAUGCAAAGAAUGCUAUUAGAAAAGUGUGACCUUCAAAAUUAUGGUGAUAGUGCAACAUUACCUAAAGGCAUAAUGAUGAAUGUCGCAAAAUAUACUCAACUGUGUCAAUAUUUAAACACAUUAACAUUAGCUGUACCCUAUAAUAUGAGAGUUAUACAUUUUGGUGCUGGUUCUGAUAAAGGAGUUGCACCAGGUACAGCUGUUUUAAGACAGUGGUUGCCUACGGGUACGCUGCUUGUCGAUUCAGAUCUUAAUGACUUUGUCUCUGAUGCAGAUUCAACUUUGAUUGGUGAUUGUGCAACUGUACAUACAGCUAAUAAAUGGGAUCUCAUUAUUAGUGAUAUGUACGACCCUAAGACUAAAAAUGUUACAAAAGAAAAUGACUCUAAAGAGGGUUUUUUCACUUACAUUUGUGGGUUUAUACAACAAAAGCUAGCUCUUGGAGGUUCCGUGGCUAUAAAGAUA |
| **PS576** | GAGCUUUGGGCUAAGCGCAACAUUAAACCAGUACCAGAGGUGAAAAUACUCAAUAAUUUGGGUGUGGACAUUGCUGCUAAUACUGUGAUCUGGGACUACAAAAGAGAUGCUCCAGCACAUAUAUCUACUAUUGGUGUUUGUUCUAUGACUGACAUAGCCAAGAAACCAACUGAAACGAUUUGUGCACCACUCACUGUCUUUUUUGAUGGUAGAGUUGAUGGUCAAGUAGACUUAUUUAGAAAUGCCCGUAAUGGUGUUCUUAUUACAGAAGGUAGUGUUAAAGGUUUACAACCAUCUGUAGGUCCCAAACAAGCUAGUCUUAAUGGAGUCACAUUAAUUGGAGAAGCCGUAAAAACACAGUUCAAUUAUUAUAAGAAAGUUGAUGGUGUUGUCCAACAAUUACCUGAAACUUACUUUACUCAGAGUAGAAAUUUACAAGAAUUUAAACCCAGGAGUCAAAUGGAAAUUGAUUUCUUAGAAUUAGCUAUGGAUGAAUUCAUUGAACGGUAUAAAUUAGAAGGCUAUGCCUUCGAACAUAUCGUUUAUGGAGAUUUUAGUCAUAGUCAGUUAGGUGGU |
| **PS100** | AUUUGACUUAGGCGACGAGCUUGGCACUGAUCCUUAUGAAGAUUUUCAAGAAAACUGGAACACUAAACAUAGCAGUGGUGUUACCCGUGAACUCAUGCG |
| **PS97** | GCUGAGGCUUCUAAGAAGCCUCGGCAAAAACGUACUGCCACUAAAGCAUACAAUGUAACACAAGCUUUCGGCAGACGUGGUCCAGAACAAACCCAAGGAAAUU |

**Supplementary Table 2: PCR primers used in this paper**

| **Name** | **Sequence (5' - 3')** |
| --- | --- |
| **N_45-419_** | F: gtgccgcgcggcagccatatgCTGCCGAACAACACCGCG  R: gtggtggtggtggtgctcgagTTACGCTTGGGTGCTATCCG |
| **N_45-364_** | F: gtgccgcgcggcagccatatgCTGCCGAACAACACCGCG  R: gtggtggtggtggtgctcgagTTACGGAAAGGTTTTGTACGCG |
| **N_45-246_** | F: gtgccgcgcggcagccatatgCTGCCGAACAACACCGCG  R: gtggtggtggtggtgctcgagTTAAACGGTTTGACCCTGTTGTT |
| **N_45-181_** | F: gtgccgcgcggcagccatatgCTGCCGAACAACACCGCG  R: gtggtggtggtggtgctcgagTTATTGGCTACCACCACGGC |
| **N_182-419_** | F: gtgccgcgcggcagccatatgGCGAGCAGCCGTAGCAGC  R: gtggtggtggtggtgctcgagTTACGCTTGGGTGCTATCCG |
| **N_182-364_** | F: gtgccgcgcggcagccatatgGCGAGCAGCCGTAGCAGC  R: gtggtggtggtggtgctcgagTTACGGAAAGGTTTTGTACGCG |
| **N_247-419_** | F: gtgccgcgcggcagccatatgACCAAAAAGAGCGCGGCG  R: gtggtggtggtggtgctcgagTTACGCTTGGGTGCTATCCG |
| **N_247-364_** | F: gtgccgcgcggcagccatatgACCAAAAAGAGCGCGGCG  R: gtggtggtggtggtgctcgagTTACGGAAAGGTTTTGTACGCG |
| **5' UTR** | F: AACTGAGATACCTACAGCGTGA  R: GATGAACACATAGGGCTGT |
| **3' UTR** | F: AACTGAGATACCTACAGCGTGA  R: GTCATTCTCCTAAGAAGCTATTA |
| **PS9** | F: AACTGAGATACCTACAGCGTGA  R: TATCTTTATAGCCACGGAACCTCCA |
| **PS576** | F: TTAATACGACTCACTATAGGGAGCTTTGGGCTAAGCGCA  R: ACCACCTAACTGACTATGAC |
| **PS100** | F: TTAATACGACTCACTATAGGATTTGACTTAGGCGACGAGCTT  R: CGCATGAGTTCACGGGTAACACCA |
| **PS97** | F: TTAATACGACTCACTATAGGGCTGAGGCTTCTAAGAAGCCTCG  R: AATTTCCTTGGGTTTGTTCTGGACCACG |
